# The Histone Deacetylase Inhibitor JAHA Down-Regulates pERK and Global DNA Methylation in MDA-MB231 Breast Cancer Cells

**DOI:** 10.3390/ma8105358

**Published:** 2015-10-16

**Authors:** Mariangela Librizzi, Roberto Chiarelli, Liana Bosco, Supojjanee Sansook, Jose M. Gascon, John Spencer, Fabio Caradonna, Claudio Luparello

**Affiliations:** 1 Department of Biological, Chemical and Pharmaceutical Sciences and Technologies (STEBICEF), Edificio 16, Università di Palermo, Viale delle Scienze, Palermo 90128, Italy; merylib@alice.it (M.L.); robertochiarelli82@libero.it (R.C.); liana.bosco@unipa.it (L.B.); fabio.caradonna@unipa.it (F.C.); 2 Department of Chemistry, School of Life Sciences, University of Sussex, Falmer, Brighton BN1 9QJ, UK; s.sansook@sussex.ac.uk (S.S.); jose.gascon@sussex.ac.uk (J.M.G.); j.spencer@sussex.ac.uk (J.S.)

**Keywords:** histone deacetylase inhibitor, extracellular-signal-regulated kinase (ERK), AKT, DNA methyltransferase (DNMT)

## Abstract

The histone deacetylase inhibitor N^1^-(ferrocenyl)-N^8^-hydroxyoctanediamide (JAHA) down-regulates extracellular-signal-regulated kinase (ERK) and its activated form in triple-negative MDA-MB231 breast cancer cells after 18 h and up to 30 h of treatment, and to a lesser extent AKT and phospho-AKT after 30 h and up to 48 h of treatment. Also, DNA methyltransferase 1 (DNMT1), 3b and, to a lesser extent, 3a, downstream ERK targets, were down-regulated already at 18 h with an increase up to 48 h of exposure. Methylation-sensitive restriction arbitrarily-primed (MeSAP) polymerase chain reaction (PCR) analysis confirmed the ability of JAHA to induce genome-wide DNA hypomethylation at 48 h of exposure. Collective data suggest that JAHA, by down-regulating phospho-ERK, impairs DNMT1 and 3b expression and ultimately DNA methylation extent, which may be related to its cytotoxic effect on this cancer cytotype.

## 1. Introduction

**Figure 1:**
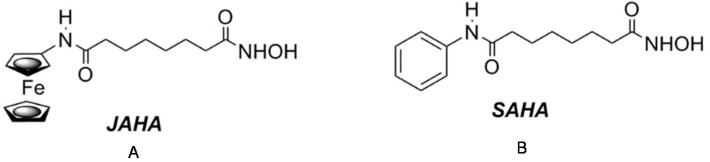
N^1^-(ferrocenyl)-N^8^-hydroxyoctanediamide (JAHA) (**A**) and suberoylanilide hydroxamic acid (SAHA) (**B**) deacetylase inhibitors (HDACis).

N^1^-(ferrocenyl)-N^8^-hydroxyoctanediamide (JAHA) is an organometallic histone deacetylase inhibitor (HDACi) analogue of suberoylanilide hydroxamic acid (SAHA), a US Food and Drug Administration-approved anticancer drug [1]. It was designed such that the three-dimensional spanning ferrocenyl group could replace the planar aryl “cap” group and act as a suitable bioisostere (Figure 1). Since JAHA’s inception, a number of metal-based analogues, including rhenium and ruthenium complexes, have appeared [2,3,4,5,6,7].

The ability of this compound to impair the growth of triple-negative, high malignant, MDA-MB231 breast tumor cells (IC_50_ at 72 h = 8.45 μM) has already been reported [8]. In particular, cell cycle perturbation, and early stage reactive oxygen species production followed by mitochondrial dysfunction and autophagy inhibition accounted for the cytotoxic effect of exposure to JAHA at its 72h-IC_50_ concentration [8], with a noticeable absence of apoptotic promotion characteristic of SAHA treatment on the same cells [9]. Here, we extended the investigation to the expression of AKT and extracellular-signal-regulated kinase (ERK) signaling, which is known to play a crucial role in tumor cell death/survival decision [10], in light of the documented ability of SAHA to deactivate both factors in different cancer cell systems [11,12,13].

## 2. Results and Discussion

**Figure 2:**
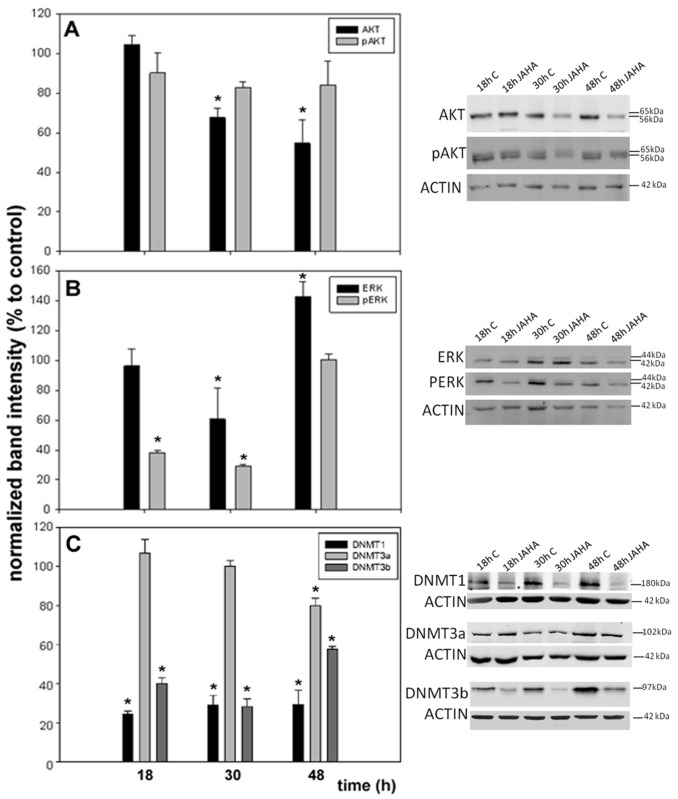
Western blot analysis of AKT, extracellular-signal-regulated kinase (ERK) and DNA methyltransferases (DNMTs). Histograms showing the accumulation of (**A**) AKT/pAKT; (**B**) ERK/pERK; and (**C**) DNMT1, 3a and 3b in JAHA-exposed MDA-MB231 cells *vs.* controls. Representative Western blots are shown on the right. The results are expressed as means ± s.e.m (standard error of the mean) of three independent Western blot experiments. * *p* < 0.05 (t-test). Statistical analysis was performed with SigmaPlot 11.0 (Systat Software Inc., San Jose, CA, USA).

In a first set of assays, MDA-MB231 cells were exposed to a 8.45 μM concentration of JAHA for 18, 30 and 48 h and Western blot analyses were performed to evaluate the accumulation of total and activated (phosphorylated) AKT and ERK1 and ERK2 isoform proteins in control and JAHA-exposed cells. As shown in Figure 2A, a decrease of total AKT down to 67.5% ± 4.8% and 54.7% ± 12% *vs.* controls was observed at 30 and 48 h of treatment with JAHA, respectively. The pAKT/total AKT ratio did not change between treated and control samples in the time lapse of the experiment. On the other hand, although exposure to 8.45 μM JAHA caused a significant decrease of the accumulation of total ERK1/2 within 30 h of culture, followed by a prominent up-regulation, a drastic reduction also in the amount of its activated forms (pERK) was observed at earlier times of treatment (18 h = 38% ± 1.4%; 30 h = 29.1% ± 1.1% *vs.* controls), as shown in Figure 2B. Also, in this case, the pERK/total ERK ratio did not change between treated and control samples in the time lapse of the experiment, suggesting that JAHA treatment impaired gene expression and not the extent of protein activation.

It is widely acknowledged that DNA methyltransferase 1 (DNMT1), DNMT3a and DNMT3b are targets for signaling through ERK pathway and they are mainly involved in variants of the enzymatic activity, *i.e.*, maintenance (DNMT1) and de novo methylation (DNMT3a and 3b) [14,15,16]. We therefore examined whether JAHA-triggered ERK1/2 deactivation could result in decreased methyltransferase expression in MDA-MB231 cells. As shown in Figure 2C, the results obtained by Western blot confirmed, at least in part, the hypothesis since JAHA treatment down-regulated DNMT1 and 3b *vs.* controls at every time point examined. In particular, the decrease of DNMT1 expression level was more prominent and steady (18 h = 24.4% ± 1.6%; 30 h = 29.1% ± 4.8%; 48 h = 29.2% ± 7.6%), whereas that of DNMT3b peaked at 30 h from exposure (18 h = 40% ± 3%; 30 h = 28.3% ± 3.8%; 48 h = 57.8% ± 1.3%). On the other hand, JAHA was not effective in modifying the expression level of DNMT3a at 18 and 30 h from exposure, whereas a late and less pronounced decrease (80% ± 3.9%) could be observed after 48 h of treatment.

**Figure 3:**
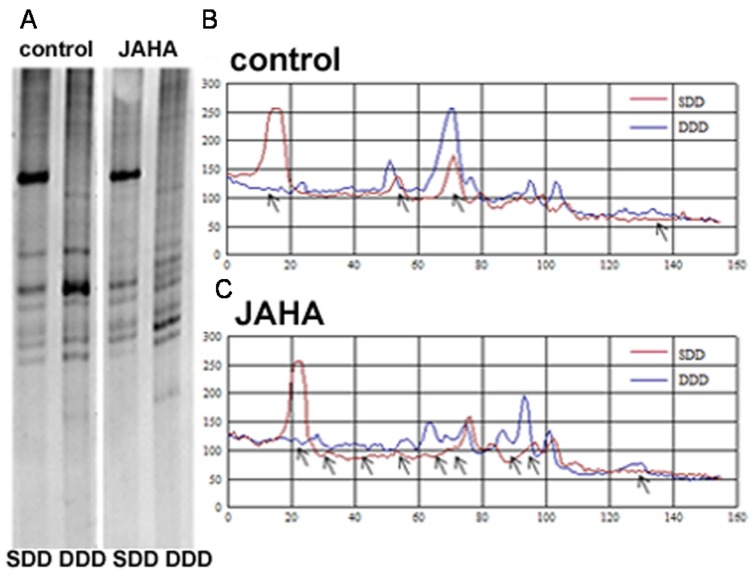
Analysis of genomewide DNA methylation status. Methylation-sensitive restriction arbitrarily-primed (MeSAP) fingerprintings (**A**) and corresponding densitometry profiles (**B**,**C**) of the matched single- (SDD in (A), red profiles in (B,C)) and double-digested DNA (DDD in (A), blue profiles in (B,C)) samples from control (**B**) and JAHA-treated (**C**) MDA-MB231 cells cultured for 48 h. The differences in the presence/absence of the peaks representing appeared/disappeared bands, or in peak heights corresponding to band intensification/attenuation (indicated by arrows in the profiles) were evaluated to compare the global methylation status. The results are representative of three independent experiments.

To confirm the observed down-regulation of DNMTs, the DNA isolated from cells grown for 18, 30 and 48 h either in control conditions or in the presence of 8.45 μM JAHA, was analyzed by methylation-sensitive arbitrarily-primed polymerase chain reaction (MeSAP-PCR) [17,18] to unveil changes induced by the drug on global methylation status of the genomic DNA. The obtained data show that 48 h-treatment with JAHA was effective in modifying the global methylation pattern of tumor cell DNA, as shown by the different number, intensity and size of the bands in the matched control and exposed samples. In particular, as shown in Figure 3, the difference in the electrophoretic patterns of single- and double-digested DNA puts in evidence an increase of unmethylated CpG-containing sites related to a hypomethylated state of the genomic DNA after exposure to JAHA. No statistically-significant difference was found at earlier times (not shown).

**Figure 4:**
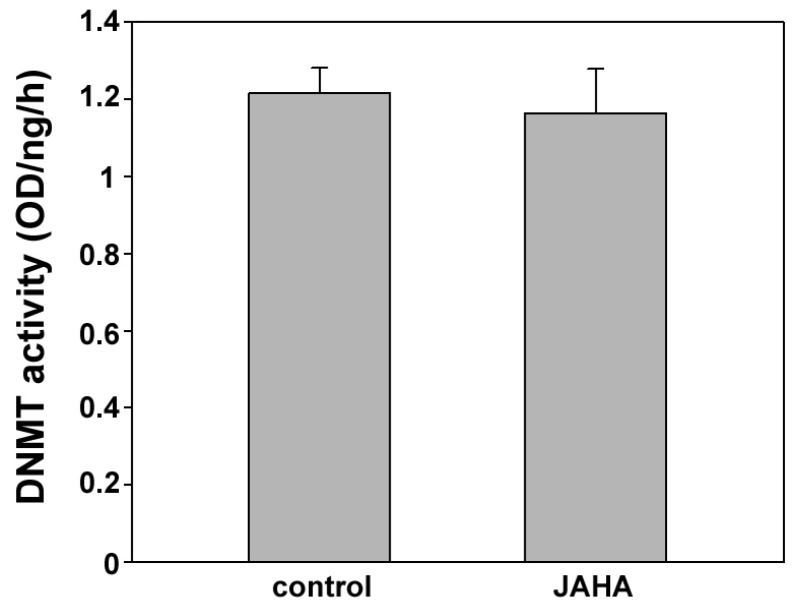
Enzyme-linked immunosorbent assay (ELISA) of DNMT activity. Histogram showing the methyltransferase activity of nuclear extracts of MDA-MB231 cells in control and JAHA-exposed conditions. The results are expressed as means ± s.e.m. of three independent ELISA assays. Statistical analysis was performed with SigmaPlot 11.0 (Systat Software Inc.). OD: Optical density.

Literature data report that HDAC1 is able to bind DNMT1 *in vivo* thereby forming a complex active on chromatin remodeling [19]. In order to ascertain whether JAHA could down-regulate global DNA methylation also by binding to this complex and interfering with DNMT action, as suggested for the HDACi trichostatin A [20], we performed an enzyme-linked immunosorbent assay (ELISA)-like DNMT inhibition test with DNMT1-containing native nuclear extract from MDA-MB231 cells in the presence or absence of JAHA. The obtained results indicated that the enzymatic activity was comparable for both control and JAHA-containing samples (Figure 4), thereby excluding a direct interaction of the drug.

## 3. Experimental Section

### 3.1. Cell Culture and JAHA Treatment

MDA-MB231 breast tumor cells were maintained in RPMI 1640 medium plus 10% foetal calf serum, 100 U/mL penicillin, 100 μg/mL streptomycin, and 2.5 mg/L amphotericin B (Life Technologies, Carlsbad, CA, USA), at 37 °C in a 5% CO_2_ atmosphere. The cells were detached from flasks with 0.05% trypsin-EDTA (ethylenediaminetetraacetic acid), counted, and plated at the necessary density for treatment after achieving 60%–80% confluency. JAHA was synthesized as reported by Spencer *et al.* [1] and dissolved at 6.5 mM concentration in dimethyl sulfoxide (DMSO) as stock solution.

### 3.2. Western Blot

Control and JAHA-treated MDA-MB231 cells were trypsinized, washed in Phosphate buffered saline (PBS), pelleted by centrifugation and lysed in a lysis buffer containing 7 M urea, 2% CHAPS, 1% IPG Buffer, 10 mM dithiothreitol (DTT), 1 mM phenylmethylsulfonyl fluoride (PMSF) and protease and phosphatases inhibitor cocktail (Sigma, St. Louis, MO, USA). The lysates were centrifuged at 1000 rpm for 1 min at 4 °C. Protein concentrations were estimated by Bradford assay (Bio-Rad, Hercules, CA, USA). Electrophoretic analysis and immunoblots were performed according to Chiarelli *et al.* [21] and Librizzi *et al.* [8] with minor modifications. Essentially, lysates of control and JAHA-treated cells were submitted to 10% sodium dodecyl sulphate-polyacrylamide gel electrophoresis (SDS-PAGE) with subsequent protein transfer onto Hybond-ECL nitrocellulose membranes (GE Healthcare, Piscataway, NJ, USA) using a Novablot semidry apparatus (Amersham Pharmacia Biotech, Piscataway, NJ, USA). Immunorevelation was performed with either rabbit polyclonal anti-Erk1/2 antibody (ab17,942, 1:500, Abcam, Cambridge, UK), anti-AKT antibody (9272, 1:750, Cell Signaling, Danvers, MA, USA), anti-p-Erk1/2 antibody (9101, 1:750, Cell Signaling), anti-p-AKT antibody (sc-7985-R, 1:750, Santa Cruz Biotechnology, Santa Cruz, CA, USA,) anti-DNMT1 antibody (SAB2106406, 1:250, Sigma), anti-DNMT3a antibody (orb228930, 1:500, Biorbyt, Cambridge, UK) or anti-DNMT3b antibody (Q-25) (sc-130,740, 1:200, Santa Cruz Biotechnology), and anti-actin antibody (A5060, 1:500, Sigma) the latter as a loading control. The secondary antibody was a horseradish peroxidase-conjugated anti-rabbit IgG antibody (W4011, Promega, Madison, WI, USA, 1:25,000). Protein bands were visualized using a Chemidoc XRS (Bio-Rad, Hercules, CA, USA) and the ImmunStar Western C Substrate Kit (Bio-Rad). The intensities of the bands of interest, evaluated with Quantity One v.4.6.6 software (Bio-Rad), were normalized for those of actin.

### 3.3. Methylation-Sensitive Arbitrarily-Primed (MeSAP)-PCR

The genomic DNA was purified from control and treated cells with the PureLinkTM Genomic DNA Kit (Life Technologies) according to manufacturers’ instructions. Two micrograms of the DNA samples were digested with 10 U of *Afa*I restriction endonuclease (Life Technologies) to generate single digested DNA (SDD) samples. The cleavage site of the enzyme is (GT*AC). Half of SDD were further treated with 5 U of *Hpa*II (Life Technologies), a methylation-sensitive restriction endonuclease unable to cut DNA if methylated cytosine is present in its recognition site (CG*GG), to generate double digested DNA samples (DDD). SDD and DDD samples were separately amplified by arbitrarily-primed PCR using two subsequent amplification cycles. In the first low stringency cycle, a permissive annealing temperature and a high salt and primer concentration were set to allow annealing of the arbitrary primer to the best matches in the template with the highest preference for all the genomic CpG sites since it is provided with a 3′ tail complementary to these sites. The first PCR cycle was performed in the presence of 500 ng of DNA, a 21-mer arbitrary primer (5′-AACTGAAGCAGTGGCCTCGCG-3′) and recombinant Taq DNA polymerase (Life Technologies), and cycle profile was 94 °C for 5 min followed by four cycles at 94 °C for 30 s, 40 °C for 60 s and 72 °C for 90 s The profile of the second high stringency cycle, performed just after the first one, was 94 °C for 1 min, followed by four cycles at 60 °C for 1 min and 72 °C for 2 min. The amplified DNA was resolved by non-denaturating 6% acrylamide-bisacrylamide (29:1 ratio) gel electrophoresis, stained with Gel Red nucleic acid gel stain (Biotium, Hayward, CA, USA), and analysed with SigmaGel v.1.0 image analysis software (SPSS, Chicago, IL, USA).

### 3.4. ELISA Assay

MDA-MB231 cells were grown in control conditions as already reported, and collected by scraping and centrifugation of the suspension. The cell pellet was re-suspended in a hypotonic buffer (20 mM Tris-HCl, pH 7.4, 10 mM NaCl, 3 mM MgCl_2_) containing 0.5% NP-40 detergent and protease inhibitor cocktail, and the nuclear proteins obtained after centrifugation of the homogenate for 10 min at 3000 rpm in the cold. The nuclear pellet was then dissolved in an extraction buffer (100 mM Tris, pH 7.4, 2 mM Na_3_VO_4_, 100 mM NaCl, 1% Triton X-100, 1 mM EDTA, 10% glycerol, 1 mM EGTA, 1 mM NaF, 0.5% deoxycholate, 20 mM Na_4_P_2_O_7_) and the nuclear proteins obtained as a supernatant after centrifugation for 30 min at 14,000 ×g in the cold and stored in aliquots at −80 °C after quantitation via Bradford assay. The DNMT-containing native nuclear extract was submitted to an ELISA-like DNMT inhibition test (EpiQuik™, Farmingdale, NY, USA, DNA Methyltransferase Activity/Inhibition Assay Kit, Epigentek, Farmingdale, NY, USA) according to manufacturer’s instructions, in the presence or absence of JAHA to check whether the drug could directly bind the enzymes and interfere with their activity. Essentially, in this kit a unique cytosine-rich DNA substrate coated on the strip wells can be methylated by DNMT enzymes transferring a methyl group to cytosine from Adomet. The methylated DNA can be recognized with an anti-5-methylcytosine antibody and its amount quantified colorimetrically through an ELISA reaction. DNMT activity (optical density (OD) (mg/h)) was calculated according to the following formula: ((sample OD − blank OD)/(sample protein × incubation time)) × 1000.

## 4. Conclusions

In conclusion, our data demonstrate that, opposed to SAHA, JAHA action on MDA-MB231 cells is addressed towards the sole deactivation of pERK1/2, leaving pAKT levels unaltered. On the other hand, the time-dependent variations in ERK1/2 level of phosphorylation are in line with those reported for SAHA-treated MDA-MB231 cells and attributable to the depletion of upstream molecules before 48 h of exposure [22]. Accumulation of total AKT appears to decrease with time whereas that of total ERK1/2 shows a U-shaped pattern with up-regulation at 48 h of exposure. This represents an additional aspect of diversity between JAHA and SAHA, whose lack of effect especially on total ERK content has been described in different cell model systems [11,12] and its biological implication remains to be determined. Our results show that JAHA inhibits mainly the accumulation of DNMT1 and DNMT3b and methyltransferase activity thereby influencing gene expression also in a manner alternative to that of histone acetylation. It is known that both DNMTs are responsible of the hypermethylation/silencing of tumor suppressor genes [23,24] and therefore it is conceivable that the DNA demethylation following JAHA treatment results in the rearrangement of the molecular landscape of transcriptional regulation and restoration of gene expression. This can ultimately account, at least in part, for the cytotoxic effect of the drug on this cancer cytotype. Characterization of the molecular basis of the different effect exerted by JAHA on DNMT3a with respect to DNMT1 and DNMT3b, and identification of the specific gene promoters targeted by the JAHA-triggered demethylation events will deserve future and more detailed investigation. These results add to a growing number of publications highlighting the use of metal-based HDACis as effective probes in cancer [5].

## References

[1] Spencer J., Amin J., Wang M., Packham G., Alwi S.S., Tizzard G.J., Coles S.J., Paranal R.M., Bradner J.E., Heightman T.D. (2011). Synthesis and biological evaluation of JAHAs: Ferrocene-based histone deacetylase inhibitors. ACS Med. Chem. Lett..

[2] Griffith D., Morgan M.P., Marmion C.J. (2009). A novel anti-cancer bifunctional platinum drug candidate with dual DNA binding and histone deacetylase inhibitory activity. Chem. Commun..

[3] Can D., Peindy N’Dongo H.W., Spingler B., Schmutz P., Raposinho P., Santos I., Alberto R. (2012). The [(Cp)M(CO)_3_] (M = Re, ^99 m^Tc) building block for imaging agents and bioinorganic probes: Perspectives and limitations. Chem. Biodivers..

[4] Spencer J., Amin J., Boddiboyena R., Packham G., Cavell B.E., Syed Alwi S.S., Paranal R.M., Heightman T.D., Wang M., Marsden B. (2012). Click JAHAs: Conformationally restricted ferrocene-based histone deacetylase inhibitors. MedChemComm.

[5] Ye R.R., Ke Z.F., Tan C.P., He L., Ji L.N., Mao Z.W. (2013). Histone-deacetylase-targeted fluorescent ruthenium(II) polypyridyl complexes as potent anticancer agents. Chemistry.

[6] De Jesús Cázares-Marinero J., Top S., Vessières A., Jaouen G. (2014). Synthesis and antiproliferative activity of hydroxyferrocifen hybrids against triple-negative breast cancer cells. Dalton Trans..

[7] Ye R.R., Tan C.P., Lin Y.N., Ji L.N., Mao Z.W. (2015). A phosphorescent rhenium(I) histone deacetylase inhibitor: Mitochondrial targeting and paraptosis induction. Chem. Commun..

[8] Librizzi M., Longo A., Chiarelli R., Amin J., Spencer J., Luparello C. (2012). Cytotoxic effects of Jay Amin hydroxamic acid (JAHA), a ferrocene-based class I histone deacetylase inhibitor, on triple-negative MDA-MB231 breast cancer cells. Chem. Res. Toxicol..

[9] Bellarosa D., Bressan A., Bigioni M., Parlani M., Maggi C.A., Binaschi M. (2012). SAHA/Vorinostat induces the expression of the CD137 receptor/ligand system and enhances apoptosis mediated by soluble CD137 receptor in a human breast cancer cell line. Int. J. Oncol..

[10] Sever R., Brugge J.S. (2015). Signal transduction in cancer. Cold Spring Harb. Perspect. Med..

[11] Bali P., Pranpat M., Swaby R., Fiskus W., Yamaguchi H., Balasis M., Rocha K., Wang H.G., Richon V., Bhalla K. (2005). Activity of suberoylanilide hydroxamic acid against human breast cancer cells with amplification of her-2. Clin. Cancer Res..

[12] Zhao Y., Yu D., Wu H., Liu H., Zhou H., Gu R., Zhang R., Zhang S., Wu G. (2014). Anticancer activity of SAHA, a potent histone deacetylase inhibitor, in NCI-H460 human large-cell lung carcinoma cells *in vitro* and *in vivo*. Int. J. Oncol..

[13] Yang B., Yu D., Liu J., Yang K., Wu G., Liu H. (2015). Antitumor activity of SAHA, a novel histone deacetylase inhibitor, against murine B cell lymphoma A20 cells *in vitro* and *in vivo*. Tumour Biol..

[14] Chang H.C., Cho C.Y., Hung W.C. (2006). Silencing of the metastasis suppressor RECK by RAS oncogene is mediated by DNA methyltransferase 3b-induced promoter methylation. Cancer Res..

[15] Chen Y., Gorelik G.J., Strickland F.M., Richardson B.C. (2010). Decreased ERK and JNK signaling contribute to gene overexpression in “senescent” CD4+CD28- T cells through epi-genetic mechanisms. J. Leukoc. Biol..

[16] Sarkar S., Abujamra A.L., Loew J.E., Forman L.W., Perrine S.P., Faller D.V. (2011). Histone deacetylase inhibitors reverse CpG methylation by regulating DNMT1 through ERK signaling. Anticancer Res..

[17] Caradonna F., Barbata G., Sciandrello G., Luparello C. (2007). Genomewide hypomethylation and PTHrP gene hypermethylation as a model for the prediction of cancer risk in rheumatoid arthritis. Novel Aspects of PTHrP Physiopathology.

[18] Naselli F., Catanzaro I., Bellavia D., Perez A., Sposito L., Caradonna F. (2014). Role and importance of polymorphisms with respect to DNA methylation for the expression of CYP2E1 enzyme. Gene.

[19] Fuks F., Burgers W.A., Brehm A., Hughes-Davies L., Kouzarides T. (2000). DNA methyltransferase Dnmt1 associates with histone deacetylase activity. Nat. Genet..

[20] Arzenani M.K., Zade A.E., Ming Y., Vijverberg S.J., Zhang Z., Khan Z., Sadique S., Kallenbach L., Hu L., Vukojević V. (2011). Genomic DNA hypomethylation by histone deacetylase inhibition implicates DNMT1 nuclear dynamics. Mol. Cell Biol..

[21] Chiarelli R., Agnello M., Roccheri M.C. (2011). Sea urchin embryos as a model system for studying autophagy induced by cadmium stress. Autophagy.

[22] Uehara N., Kanematsu S., Miki H., Yoshizawa K., Tsubura A. (2012). Requirement of p38 MAPK for a cell-death pathway triggered by vorinostat in MDA-MB-231 human breast cancer cells. Cancer Lett..

[23] Jacinto F., Ballestar E., Ropero S., Esteller M. (2007). Discovery of epigenetically silenced genes by methylated DNA immunoprecipitation in colon cancer cells. Cancer Res..

[24] Subramaniam D., Thombre R., Dhar A., Anant S. (2014). DNA methyltransferases: A novel target for prevention and therapy. Front. Oncol..

